# Brain-Derived GLP-1—Understanding the Physiological Function and Anti-obesity Potential of Preproglucagon Neurons

**DOI:** 10.1210/endocr/bqaf169

**Published:** 2025-11-12

**Authors:** Stefan Trapp, Cecilia Skoug

**Affiliations:** UCL Centre for Cardiovascular and Metabolic Neuroscience, Department of Neuroscience, Physiology & Pharmacology, University College London, London WC1E 6DE, UK; UCL Centre for Cardiovascular and Metabolic Neuroscience, Department of Neuroscience, Physiology & Pharmacology, University College London, London WC1E 6DE, UK

**Keywords:** GCG, brainstem, PPG, food intake, obesity, transgenic

## Abstract

Glucagon-like peptide-1 (GLP-1) is produced within the central nervous system (CNS) by preproglucagon (PPG) neurons. This brain-derived GLP-1, rather than that released from the gut, is the physiological agonist for brain GLP-1 receptors (GLP-1Rs). With brain GLP-1Rs being a major target for eating suppression, understanding the physiology and the translational potential of PPG neurons is of pivotal importance, particularly since PPG neuron activation is also strongly associated with stress. This review critically summarizes the current knowledge of PPG neuron anatomy, physiology, and molecular makeup together with insight into the relevant research tools, and consideration of the different PPG neuron populations within the CNS, to provide an appraisal of the potential of these neurons as drug targets and the associated risks and benefits.

The most extensively studied population of preproglucagon (PPG) neurons comprises a few thousand neurons located in the lower brainstem. As their name indicates, these cells transcribe the product of the glucagon (*Gcg*) gene. Proglucagon is then processed posttranslationally to create glucagon-like peptide-1 and -2 (GLP-1/2) as well as glicentin that is further processed to oxyntomodulin (1). Often, immunocytochemical detection of GLP-1 (or GLP-2) is used to identify PPG neurons in brain tissue (2) and consequently they are also called GLP-1 neurons. While they have been studied most intensively in the mouse and rat, this brainstem population of PPG neurons has been identified in other mammals, including humans (3, 4), and in nonmammalian vertebrates, namely birds (5, 6). The observation that this central nervous system (CNS) source of GLP-1 is conserved, at least in vertebrates, suggests functional importance. However, this functional importance has not been clarified unequivocally to date. Interestingly, in addition to the global preservation of these cells across species, there are also reports of functional differences, primarily between rat and mouse, for example (7), the only two species with a reasonable number of functional studies that can be compared. Hence, it is currently unclear whether functional aspects differ more between nonrodent species.

## Neuroanatomy

Brainstem PPG neurons and their projections were first described as GLP-1 immunoreactive neurons in rat in the late 1980s (8). Individual cell bodies were found in the caudal nucleus tractus solitarii (NTS) and in the intermediate reticular nucleus (IRT), while axons were detected in multiple brain regions, with the highest abundance in the hypothalamus (2, 8). Immunostaining of axon terminals with a GLP-1 antibody clearly demonstrated that GLP-1 neurons store GLP-1 as a neurotransmitter or neuromodulator at the release sites for the peptide. This finding was later confirmed by electron microscopy localizing GLP-1 to dense core vesicles (9), and also by the observation that large amounts of active GLP-1 are located in the hypothalamus, which has dense innervation from these neurons but contains no PPG cell bodies (10).

Expression of preproglucagon in brainstem cell bodies was confirmed by in situ hybridization (11), and generation of *Gcg*-transgenic mice expressing yellow fluorescent protein (YFP) under control of the glucagon promoter allowed ever more detailed analysis of the projection pattern of these neurons (12, 13). For example, these transgenic mice enabled the discovery that a substantial proportion of PPG neurons project into the spinal cord (14). In addition to the populations in NTS and IRT, small numbers of PPG neurons were also found along the midline ventral from the hypoglossal nucleus in a position overlapping with the raphe obscurus. These were also detected by in situ hybridization in the rat (11) and recently confirmed in a new transgenic rat model (15).

Additionally, the mouse expressing YFP under the glucagon promoter revealed PPG cell bodies in the olfactory bulb, the piriform cortex, and the lumbar-sacral spinal cord, but their physiological function remains unclear to date. While those in the spinal cord (14) have not been described in other species, PPG neurons in piriform cortex and olfactory bulb have also been reported in the rat (11, 15). Furthermore, functional characterization of the olfactory bulb PPG neurons has begun in the mouse (16-19). These studies suggest that olfactory bulb PPG neurons, which are located in the granule cell layer, are either local interneurons or short axon cells that do not have far-reaching projections outside the olfactory bulb.

There is consequently a consensus that at least the vast majority of GLP-1–containing axonal projections originate from the brainstem population of PPG neurons. Several lines of evidence support this notion. First, injection of retrograde tracers into various forebrain areas (20-22), as well as injection into the spinal cord (14), labels brainstem PPG neurons. Second, viral transduction of brainstem PPG neurons with fluorescent markers labels axons in the forebrain (23, 24). Third, knockdown of PPG expression in the NTS reduced GLP-1 immunoreactivity in the paraventricular nucleus of the hypothalamus (PVN) in the rat (25), and finally, virally mediated selective ablation of PPG neurons in the NTS in the mouse significantly reduced the amount of active GLP-1 detected not only in the brainstem, but also in the hypothalamus and spinal cord (10). Based on these observations it seems very plausible that the widespread PPG axon projections throughout most of the brain originate from the brainstem.

The PPG neuron cell bodies in NTS and IRT are in spaces partially overlapping with the location of dorsal vagal neurons and parasympathetic nucleus ambiguous neurons, respectively (12). However, unlike these efferent vagal neurons, axons from PPG neurons do not leave the CNS (14). Whether their physical location close to these vagal efferents is of functional significance is presently unclear; however, several studies in rat suggest that dorsal vagal neurons are sensitive to GLP-1 (26, 27). It is also unclear whether NTS and IRT populations of PPG neurons form distinct groups or a continuum along the arc from NTS to ventrolateral IRT. Retrograde tracing from projection targets such as the dorsomedial hypothalamus or the spinal cord label both NTS and IRT PPG neurons (14, 21, 24). Overall, to date there is no indication of distinct populations.

However, the neuroanatomy of the central GLP-1 system is highly suggestive of an organization by which GLP-1 is stored within axon terminals or varicosities along the length of axons, with release of GLP-1 into the brain parenchyma synaptically or perisynaptically in response to electrical signals within each individual axon. Thus, GLP-1 released at one specific brain site could have a different effect than GLP-1 released at a different site. On the other hand, Card et al (28) showed close appositions between NTS PPG neurons in the mouse suggestive of potential crosstalk between individual PPG neurons. Additionally, another study in the rat demonstrated that PPG neurons innervate the paraventricular nucleus of the thalamus (PVT), the PVN, and the bed nucleus of the stria terminalis (BNST) collectively, and in light of the anatomical connectivity of NTS PPG neurons described before, it proposed that PPG neurons form a tightly coordinated network in which activation of a subset of neurons will lead to concerted activation of the entire population (24). However, this proposed model of synchronized action is based only on anatomical observations and it is currently unclear whether brainstem PPG neurons form a homogeneous population that produces synchronous effects at diverse projection targets or a highly diverse population of individual PPG neurons that have divergent functions. These functional considerations are discussed in the section Physiological Role of Preproglucagon Neurons.

## Transgenic Animal Models as Essential Tools to Investigate and Understand Preproglucagon Neurons

Many of the investigations into the physiology of PPG neurons would not have been possible without the use of transgenic animal models. While these are invaluable, essential tools, it is important to be aware of both the opportunities and the limitations that these present.

The first mouse model that allowed identification of PPG neurons by fluorescent marker was the Glu-Venus mouse (29). In this model, the YFP Venus was introduced as a simple transgene consisting of a bacterial artificial chromosome containing the *Gcg* promoter, followed by Venus in an open reading frame. The transgene was randomly inserted into the mouse genome and the copy number was undefined. With Venus under the direct control of the strong *Gcg* promoter, highly fluorescent PPG neurons were detected without the requirement of amplification with immunofluorescence (30). The resulting pattern of fluorescently labeled cell bodies primarily in the lower brainstem as well as axon terminals in various forebrain regions (13), including the limbic system (23), matched well the previously reported distribution of GLP-1 immunoreactivity in the rat (2). Additionally, the distribution of Venus through all cell compartments enabled visualization of the entire cells, including dendritic trees and axons in passage (13), which are not detected by GLP-1 antibody staining, because GLP-1 is mainly found in cell bodies and axon terminals. The untargeted genetic approach ensured that the native *Gcg* gene in these mice was not affected but came with the risk that random insertion could disrupt another important gene. Subsequently, two more mouse models were generated independently with a similar approach to create mice that express cre-recombinase under control of the *Gcg* promoter (31, 32). These mice allowed targeted expression of foreign genes, such as DREADD receptors or channelrhodopsin, selectively in PPG neurons by stereotaxic injection of adeno-associated viruses that encode these proteins in a Cre-dependent fashion. This enabled functional manipulation of PPG neurons to elucidate their function, as discussed in more detail next (10, 32). While no adverse effects have been reported for these strains using random insertion, there could potentially be issues. This would be avoided by a targeted approach such as a knock-in. In fact, another mouse line was created with a targeted approach where improved Cre recombinase was inserted into exon 2 of the *Gcg* gene (33). Interestingly, however, in this mouse line the *Gcg* gene locus was accidentally duplicated, leading to the targeted allele possessing both the transgene and the unaltered wild-type *Gcg* gene. These mice have been used to assess NTS PPG neuron activity in vivo with Ca^2+^ fiber photometry and to optogenetically stimulate the same cells (34).

The latest addition to the transgenic animal toolbox available to study PPG neurons is a transgenic rat (15). The advantages of this addition are not only to confirm PPG action/neuroanatomy in a different rodent model, but rat models have distinct advantages in research in general due to the similarity in genetics to humans, the advantages of a larger size in some experimental techniques, and the difference in baseline behavior compared to mice (35).

This rat model was generated with CRISPR technology to target an internal ribosome entry site (IRES)-Cre construct to the end of the open reading frame of the *Gcg* gene. By targeting the *Gcg* gene, this approach avoids the potential complications caused by random insertion of the transgene. The use of an IRES construct ensured that the Cre-recombinase is translated independently of the proglucagon peptide and should not interfere with the expression of the native *Gcg* gene product. Crossing this rat with a tdTomato reporter strain revealed an expression pattern for the tdTomato fluorescence very similar to that reported for the cre-reporter mice, but GLP-1 levels in the blood and PPG messenger RNA (mRNA) levels in the brain were severely reduced particularly in homozygous rats (15), suggesting that targeting the construct to the native *Gcg* gene affected transcription and translation level despite the use of an IRES construct. As such limitations were not observed in the knockin mouse (34), the accidental duplication of the allele in the mouse might have been rather beneficial for the overall phenotype.

These observations highlight both the opportunities as well as the limitations of different transgenic approaches. First, activity of the *Gcg* gene within the CNS varies during development, but if cre-recombination is used to switch on expression of a reporter gene by crossing the cre-expressing mouse strain with a floxed reporter mouse strain, then the reporter is permanently expressed in all cells that exhibited Cre expression at some point during development (36). Hence, reporter expression in a specific neuron in an adult mouse does not necessarily mean that this neuron is expressing GLP-1 at the point in time when a study is performed. In accordance with this caveat, more widespread expression of the reporter has been reported for the *Gcg*-cre mice compared to the expression of Venus directly under the *Gcg* promoter (32). In both the rat and mouse *Gcg*-cre model, this has been shown by reporter expression in areas such as the paraflocculus of the cerebellum, inferior colliculus, basolateral amygdala, and orbital cortex (15). Investigating the areas further, the basolateral amygdala was confirmed to show *Gcg* expression in neonates, but not adult-age rats (15). Second, there might be the question of physiologically relevant expression levels. If an individual cell aberrantly expresses a few molecules of GLP-1 or glucagon, this has no physiological consequences. If the cell however expresses a few molecules of Cre-recombinase, this is sufficient to elicit cre-dependent expression of virally or transgenically delivered exogenous genes. Thus Cre-driver mice should always be used with caution to ensure that those cells that are manipulated functionally in a cre-dependent fashion are indeed those that comprise the physiologically relevant population of cells intended to be targeted.

## Preproglucagon Neurons of the Olfactory Bulb

With the development of transgenic models, the capability of studying PPG neurons increased substantially, and this also provided the impetus to investigate PPG neurons described in brain regions beyond the lower brainstem. While the presence of PPG mRNA in the olfactory bulb of the rat was first reported 25 years ago (11), attempts to functionally study these cells began only 10 years ago with ex vivo studies demonstrating the presence of a microcircuit within the olfactory bulb, where GLP-1 is produced by short axon cells in the granule cell layer and mitral cells being responsive to GLP-1 (16, 17). Importantly, these functional studies demonstrated that olfactory bulb PPG neurons are glutamatergic like their counterparts in the lower brainstem, and not GABAergic as was expected for interneurons located within the olfactory bulb granule cell layer. Building on these studies, more recently a couple of studies by Montaner et al (18, 19) demonstrated with short hairpin RNA knockdown of GLP-1 expression in the olfactory bulb that GLP-1 signaling from these PPG neurons is necessary for odor-evoked cephalic phase insulin release and that this cephalic response was dependent on the sympathetic nervous system, rather than the vagal nerve. Additionally, they demonstrated that local administration of the GLP-1 receptor (GLP-1R) antagonist exendin-9 extended the time taken for mice to find buried food, indicating that GLP-1 signaling in the olfactory bulb increases either the ability to find food or the interest in food (18). A striking finding about PPG neurons from these studies is the observation that GLP-1 in the olfactory bulb increases rather than decreases the interest in, or ability to detect, food. In the brainstem and in relation to GLP-1 receptor agonists (GLP-1RAs), it is usually the postprandial situation that is considered, whilst here it is a preprandial role that GLP-1 fulfills. Regarding the effects on insulin release, it clearly works like peripheral GLP-1. These observations raise the question whether it would be counterproductive if “postprandial” GLP-1 could spill over from the circulation or from brainstem PPG neuron release into the olfactory bulb: Would this enhance, rather than suppress, food intake?

**Figure 1.**
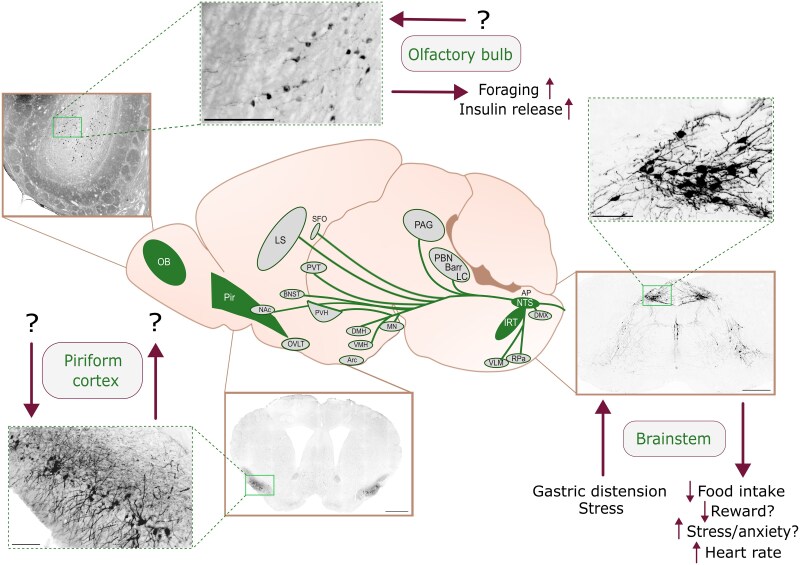
Anatomical distribution of the preproglucagon (PPG) neuron populations, their known projections, regulation, and functions. The cell bodies of PPG neurons are located in the olfactory bulb (OB, upper left), the piriform cortex (Pir, lower left), and the caudal nucleus tractus solitarii (NTS) and intermediate reticular nucleus (IRT) of the brainstem (right), here visualized in the Glu-Venus mouse model (13, 29, 30). In the OB, PPG neurons reside in the granule cell layer, and when stimulated increase foraging in mice. Little is known about the PPG neurons in the Pir; neither stimulus nor outputs have been characterized yet. Most PPG neurons are found in the lower brainstem from where they project to a plethora of forebrain areas. This population is activated by gastric distension and various stimulus of stress, with hypophagia as the main physiological outcome. While the mechanisms driving the hypophagia have not been fully elucidated, it has been suggested that activation of these PPG neurons suppresses reward circuitry contributing to the reduction in food intake. The involvement of brainstem PPG neurons, particularly NTS PPG neurons, has been suggested to induce a stress response, and at least an increase in heart rate has been demonstrated on direct activation of these neurons. AP, area postrema; Arc, arcuate nucleus; Barr, Barrington nucleus; BNST, bed nucleus of the stria terminalis; DMH, dorsomedial hypothalamus; DMX, dorsal motor nucleus of the vagus; IRT, intermediate reticular nucleus; LC, locus coeruleus; LS, lateral septum; MN, mammillary nucleus; NAc, nucleus accumbens; NTS, nucleus tractus solitarii; OB, olfactory bulb; OVLT, organum vasculosum of the lamina terminalis; PAG, periaqueductal gray; PBN, parabrachial nucleus; Pir, piriform cortex; PVH, paraventricular nucleus of the hypothalamus; PVT, paraventricular thalamus; RPa, raphe pallidus; SFO, subfornical organ; VLM, ventrolateral medulla; VMH, ventromedial hypothalamus. Scale bars: 250 μm for main images; 40 μm for inserts.

One could argue that this emphasizes the need to keep the different GLP-1 systems in the body separate (37, 38). If it is assumed that the different PPG neuron populations (Fig 1), namely an olfactory bulb system, a piriform cortex system, and the main system originating from the lower brainstem, reflect 3 distinct CNS GLP-1 systems, the requirement arises to keep these separated. Working on the assumption that GLP-1 is released synaptically within each of these systems and that its spread is limited to the synaptic and perisynaptic space, this separation is easily achieved. However, GLP-1 is both a neurotransmitter produced within the brain and a peptide hormone secreted as an incretin from the L cells of the gut epithelium into the bloodstream and potentially distributed throughout the body. Hence, to ensure temporally and spatially precise signaling in different projection targets of neuron populations within the CNS, it is essential that the central GLP-1 system be isolated from hormonally acting circulating GLP-1. This has recently been demonstrated in the mouse and has been discussed in detail elsewhere (37, 38). In fact, that study not only indicated a clear separation between the targets for gut-derived GLP-1 and GLP-1 from PPG neurons but also suggested that systemically administered exogenous GLP-1RAs act on targets different from PPG neurons to reduce food intake. Whether this separation also means that GLP-1RAs categorically do not cross the blood-brain barrier (BBB) has not been comprehensively clarified and its discussion, while important, is beyond the scope of this review.

Since it seems to be imperative to prevent spillover of GLP-1 between the olfactory bulb and the brainstem PPG neurons, would a highly brain-permeant GLP-1RA be counterproductive? We will come back to this question after focusing on the putative physiological role(s) of brainstem PPG neurons.

## Physiological Role of Preproglucagon Neurons

The precise physiological function of PPG neurons has remained largely unresolved to date and has often been shaped by our preconceptions and assumptions rather than unbiased observation. We are also regularly conflating the physiological role with the potentially achievable effect on pharmacological or chemogenetic stimulation.

A longstanding question in GLP-1 physiology was that of whether PPG neurons are simply an “extension” of the gut GLP-1 system into the brain. This hypothesis has been refuted by several lines of independent evidence. First, PPG neurons themselves do not express GLP-1Rs and do not respond to GLP-1 (28, 30). Additionally, systemic application of GLP-1RAs does not elicit cFos in PPG neurons (38, 39), and GLP-1–expressing vagal afferents do not innervate PPG neurons (38). Furthermore, Holt et al in 2019 (10) demonstrated that ablation of NTS PPG neurons strongly reduces the amount of GLP-1 detectable in the brainstem, hypothalamus, and spinal cord. Finally, concomitant systemic administration of a GLP-1RA and chemogenetic activation of PPG neurons produces stronger hypophagia than either treatment alone (38). Thus, PPG neurons and peripheral GLP-1 do not appear to be functionally linked. This concept has been discussed in detail recently (37) and will not be revisited here.

Studies investigating the physiology of PPG neurons fall into two groups. They either address the question of what stimuli activate PPG neurons or they ask what the consequence of PPG neuron activation is. The most commonly reported consequence of PPG neuron activation is a reduction in food intake, and the most commonly reported stimuli of PPG neurons are gastric or systemic satiety or satiation signals, both electrical and hormonal, and stress, both visceral and physical/psychological (30, 34, 40-43).

Until transgenic mouse models allowed unequivocal identification as well as manipulation of the brainstem PPG neurons, only post hoc analysis of cFos expression, as a surrogate marker for neuronal activity, coupled with GLP-1 immunocytochemistry provided insight into what stimuli activate these cells (40). Direct recording of the electrical activity of PPG neurons became feasible only with transgenic mice that fluorescently labeled PPG neurons, enabling their identification in brain(stem) slices for patch-clamp recordings (30, 41). Another line of investigation has used the intracranial administration of GLP-1 (RAs and antagonists) to glimpse an insight into the role of GLP-1 within the CNS. Particularly, brain administration of antagonist only has demonstrated that native GLP-1 has physiological effects within the brain (20, 44-47). The various effects of GLP-1RAs in multiple parts of the CNS have been reviewed comprehensively (48, 49) and will thus only be touched on in this review. Activation of GLP-1Rs inside the BBB could potentially replicate either the action of gut-derived GLP-1 gaining access to the brain, or the action of PPG neurons. However, various studies have indicated that postprandial gut-derived GLP-1 is extremely unlikely to reach the brain (50-52), making it most likely that GLP-1Rs inside the BBB are the physiological target of PPG neurons rather than GLP-1 released from enteroendocrine L cells. This view is further substantiated by the observation that knockdown of gut GLP-1 affects glucose tolerance but not food intake and body weight (53). However, this does not preclude the possibility that systemic administration of pharmacological doses of GLP-1RAs, particularly under pathophysiological conditions that compromise the BBB, might be able to elicit some of the responses attributed physiologically to PPG neuron activation. Here, we will first focus on what stimuli activate PPG neurons and then examine the consequences of PPG neuron activation, both at the physiological and pharmacological level.

### Stimuli that Activate Preproglucagon Neurons

It has been suggested that PPG neurons are primarily activated by adverse effects, be these stress, severe gastric distension, or enteric malaise, rather than physiological signals. For example, a study comparing cFos expression in PPG neurons and catecholaminergic A2 neurons in response to voluntary meals found only the very largest meals elicited cFos in PPG neurons, while cFos in A2 neurons tracked meal size (54). A second study with meal-entrained rats demonstrated a strong positive correlation between the amount of liquid diet consumed and the percentage of cFos-expressing PPG neurons (42). Similarly, a study in the mouse found that only unusually large meals increased subsequent food intake after chemogenetic inhibition of NTS PPG neurons, suggesting that normal meals do not activate NTS PPG neurons sufficiently to suppress subsequent food intake (10). While these studies suggest that only unusually large intake stimulates PPG neurons, a recent study in mice that monitored Ca^2+^ dynamics of individual PPG neurons in vivo demonstrated an initial response of these cells to food intake within seconds. This was followed by increasing activity over minutes, reflecting the tracking of cumulative intake, and is suggestive of a physiological response to food intake (34). Similarly, when liquid food was infused directly into the stomach at a rate of 1 mL in 10 minutes, the Ca^2+^ signal in PPG neurons ramped up over this period rather than occurring only once strong gastric distension was achieved with the entire volume. Also, interestingly, infusion of 1 mL of saline did not elicit a response despite its volume. While these observations strongly suggest that the PPG neuron activation is elicited by gastric signaling, and not only under drastic gastric distension, this does not seem to be gastric distension alone, though infusion of 1 mL of air did produce some response. It is currently unclear how these observations can be reconciled and whether they are due to different experimental paradigms in these studies. Possibly only strong stimulation reaches the threshold for cFos expression while smaller stimulation already elicits measurable Ca^2+^ signals.

It might be worth taking a closer look at the activation of PPG neurons by gastric distension. While this aligns with the old observation that PPG neurons receive inputs from vagal afferents (30), recent data might suggest subtle nuances in this picture. First, in rats, only strong gastric distension activated these neurons, while moderate distension activated catecholaminergic NTS neurons (54). Second, GLP-1R–expressing vagal afferents are not a significant input to PPG neurons, despite these neurons being shown to carry gastric distension signals (38). Third, a recent study has demonstrated that ilial GLP-1 or the systemically administered GLP-1RA exendin-4 induce gastric distension (52), while the same compound and other GLP-1RAs fail to induce cFos in PPG neurons (39). While it could still be argued that exendin-4 might not produce sufficient gastric distension to induce PPG neuron activation, it might be pertinent to consider a different interpretation. Oxytocin-receptor expressing vagal afferents have been shown to be a strong vagal input to PPG neurons (38). Additionally, oxytocin-induced hypophagia was strongly attenuated when NTS PPG neurons were ablated (38), indicating the physiological importance of this pathway. Considering that oxytocin receptor–expressing vagal afferents receive inputs from the duodenum, rather than the stomach (55), this might suggest that PPG neurons are not activated by gastric distension, but rather by duodenal extension. The findings from strong gastric distension might be explained by the suggestion that extreme gastric distension actually causes duodenal distension, which is then signaled to the PPG neurons. In this context it is interesting to note that systemically administered exendin-4 delays gastric emptying, which would reduce any duodenal distension, and this effect is prevented by knockdown of vagal-afferent GLP-1Rs (56).

Restraint stress–induced hypophagia requires endogenous GLP-1 signaling within the brain (23, 45, 46, 48), and subsequently a few studies have demonstrated directly a strong activation of PPG neurons by restraint stress (10, 57). This is not linked to (postprandial) vagal-afferent signals and indicates that PPG neurons must receive input from other sources as well. Holt et al (58) revealed a multitude of synaptic inputs to PPG neurons from throughout the CNS. They demonstrated that PPG neurons in the caudal NTS receive direct descending inputs from forebrain regions implicated in stress processing, including the PVN. Additionally, Leon et al (57) identified serotonin signaling via 5-HT_2C_ and 5-HT_3_ receptors as necessary for cFos expression in PPG neurons in response to LiCl or restraint stress in rat. Interestingly, mouse PPG neurons do not respond to 5-HT_3_ receptor agonists in brain slices ex vivo but are responsive to 5-HT_2C_ and 5-HT_1A_ receptor activation (59, 60), a finding that was further validated by the observation that the 5-HT_2C_ receptor agonist lorcaserin elicits cFos expression in mouse PPG neurons (60). These studies indicate both parallels as well as differences between the rat and mouse for brainstem serotonin signaling.

As stated earlier, systemic and psychogenic stressors activate brainstem PPG neurons and are hypothesized to activate the hypothalamic-pituitary-adrenal (HPA) axis (61), based on increases both of plasma corticosterone and adrenocorticotropin after intracerebroventricular injection of GLP-1 in the rat (62). Zhang et al (63) demonstrated this involvement from a different angle, in which a decrease in *Gcg* mRNA but an increase of *Gcg* heteronuclear RNA in the NTS and reduced GLP-1 immunoreactivity in the PVN was observed in response to restraint stress and to LiCl, both of which were accompanied by an increase in plasma corticosterone. Injection of corticosterone also led to downregulation of PPG mRNA, suggesting that the lower PPG mRNA amount is due to glucocorticoid secretion. The concurrent increase of heteronuclear RNA, an indicator of recent gene transcription, and decrease of mRNA suggests that the loss of mRNA is not due to reduced transcription, but rather a feedback loop to regulate GLP-1 availability after rapid PPG neuron activation to limit the GLP-1–induced activation of the HPA axis (63). These data suggest that PPG neurons are not only stimulated by the acute stress response but also limit the response. Whether activation of PPG neurons is dampened during chronic stress remains to be investigated.

Although the aforementioned study did observe a decrease in *Gcg* mRNA with the injection of LiCl, there was no measurement of actual PPG neuron activation with the stimulus (63). It has previously been reported that pharmacological stressors such as lipopolysaccharide and LiCl elicit a cFos response in PPG neurons when tested in rats (40). However, a recent study recording Ca^2+^ dynamics of PPG neurons with fiber photometry failed to observe immediate activation of PPG neurons with LiCl and lipopolysaccharide (34). This discrepancy raises the question of whether there is a context dependence in PPG engagement during stress, or alternatively whether activation of PPG neurons by stress differs between the rat and mouse.

Additionally, electrophysiological recordings from PPG neurons ex vivo have demonstrated their direct activation by leptin, glutamate, vagal afferent stimulation, and presynaptic modulation of glutamatergic inputs by cholecystokinin (CCK-8) and epinephrine, but no direct or indirect effects of GLP-1, PYY3-36, ghrelin, and melanotan II (30, 41). Similarly, Ca^2+^ recordings from PPG neurons in ex vivo brainstem slices demonstrated responsiveness to oxytocin (38), interleukin-6 (64), leptin, CCK-8, and 5-HT_2C_ receptor agonists (59), but not GLP-1 (28) or 5-HT_3_ receptor agonists (59). Further, systemic administration of peptides linked to the physiological regulation of satiety or satiation, such as CCK, elicited cFos immunoreactivity in PPG neurons (40). A further step change in the abilities to interrogate the regulation of PPG neuron activity was the demonstration that fiber photometry recordings of NTS PPG neurons in awake mice are feasible, despite the amount of movement in the caudal NTS. This first study provided exciting insight into the time course of activation by ingestive signals as well as confirmation of the pharmacology determined by the earlier ex vivo studies (34). It will be exciting to see whether this can be pushed technically even further to enable endoscopic recordings from individual PPG neurons in vivo.

In summary, PPG neurons receive diverse inputs ranging from signals reflecting adiposity, via satiation signals to those indicating malaise or stress, as well as multiple inputs from the forebrain, suggesting that PPG neurons balance various inputs to generate an output that determines the appropriate level of food intake for the given situation. As such, these neurons do not seem to determine energy balance but are informed by energy balance and other inputs to optimize decisions to ensure survival. For example, in a situation of sufficient energy reserves and a full stomach, it is not necessary to take the risk of seeking food, and hence their activity suppresses food intake, whereas in negative energy balance their activity is suppressed and circuits that promote energy intake are not overridden by the activity of PPG neurons (65). Consequently, neuronal GLP-1 knockout has no substantial effect on body weight, particularly in ad libitum–fed laboratory animals (10, 66), because the homeostatic regulation of food intake remains intact. In contrast, pharmacological activation of the central GLP-1 system has the capacity to strongly suppress food intake (10, 32, 38, 67). This is also seen with systemically administered GLP-1RAs that are highly effective in reducing body weight, while removal of the receptors has minimal effect on body weight (68).

Such physiological regulation would make the PPG neurons highly desirable as a target for weight-loss medication, unless their activation is linked to effects beyond food intake suppression that is detrimental to health. To evaluate this, the next section focuses on the effects elicited by pharmacological, chemogenetic, or optogenetic stimulation of PPG neurons.

### Effects Elicited by Preproglucagon Neuron Activation

When considering effects of PPG neuron stimulation, it is critical to ensure that the observed physiological effects in response to, for example, pharmacological stimulation of PPG neurons are indeed downstream effects and not due to the substance used also activating parallel, PPG neuron–independent pathways that cause the observed effects. For example, while stress undoubtedly activates PPG neurons, it is less clear which of the consequences of stress, such as hypophagia, activation of the HPA axis, or tachycardia, are dependent on PPG neuron activation. In pharmacological experiments, this is usually addressed by demonstrating that the response is blocked by administration of a GLP-1R antagonist. However, this has two potential caveats: First, PPG neurons are also glutamatergic—therefore, some of their physiological effects could be mediated by glutamatergic transmission; second, the GLP-1R antagonist would also block potential effects mediated by independent release of GLP-1 from enteroendocrine L cells; and third, it is doubtful whether the antagonist when given systemically would cross the BBB (69). Consequently, the most reliable approach to ascertain functions selectively fulfilled by PPG neuron activation is their chemogenetic or optogenetic activation. Additionally, selective chemogenetic or optogenetic inhibition or ablation of the PPG neurons enables determination of which physiological or pharmacological effects require functional PPG neurons.

Acute chemogenetic activation of brainstem PPG neurons has consistently yielded a substantial reduction in food intake both in mice and rats (10, 15, 32, 38) and so has optogenetic stimulation of PPG neuron terminals in the PVN (67). This study additionally demonstrated that the optogenetic stimulation of these PPG neuron terminals reduced food intake in a GLP-1–dependent manner because it persisted in vGlut2-knockout mice, thus confirming that the cotransmitter glutamate is not the driver for reduced food intake. Finally, that study revealed an increase in food intake on optogenetic inhibition of PPG neuron terminals in the PVN, suggesting ongoing endogenous release of GLP-1 in the PVN under their experimental conditions (67). This is interesting because chemogenetic inhibition or ablation of NTS PPG neurons produced hyperphagia only when either restraint stress or an abnormally large meal had activated these PPG neurons (10). In contrast to the consistent effect on food intake, a glucose-lowering effect of chemogenetic PPG neuron stimulation was observed in one study (70) but not in another (10). Finally, chemogenetic activation of NTS PPG neurons increases heart rate both in anesthetized and awake mice (39). This could indicate an involvement in a fight-and-flight response.

A number of studies link GLP-1/GLP-1R and stress response (62, 71), but there are few data to demonstrate direct involvement of PPG neurons. A recent study employing chemogenetic activation of PPG neurons resulted in female mice reducing both the time spent in the center of the open field arena and the total distance traveled (72). These results were interpreted as a stress or anxiety-like response and this interpretation was further supported in an acoustic startle test, although in a sex-independent manner. In contrast, Gaykema et al (32) in 2017 failed to observe a difference in stress response measured by open field or elevated plus maze and also did not observe an increase in plasma corticosterone in response to chemogenetic activation of NTS PPG neurons. However, that study was performed in male rats, so no sex-dependent effect would have been noted.

Although evidence supports a modulatory role for PPG neurons and GLP-1 in stress-related behavior, several questions remain to be answered. First, while GLP1R activation induces anxiety-like behaviors and activates the HPA axis (73-76), it is still unclear to what extent endogenous GLP-1 release from PPG neurons drives these effects under physiological conditions. Second, it is also unclear what drives the difference in the response in females compared to males and whether there is a stressor-specific PPG neuron response (with potential different outcome downstream)?

Consequently, one might ask the question whether these are all individual aspects of a concerted physiological response that involves activation of all PPG neurons, or are there a variety of PPG neuron groups with different projection patterns and different inputs that reflects a range of physiological functions that are controlled/modulated by central GLP-1?

## Are There Preproglucagon Neuron Subpopulations?

Release of GLP-1 at one place and time within the CNS might reduce food intake in response to peripheral signals such as gastric distension, whereas GLP-1 release at a different site, such as onto sympathetic preganglionics in the spinal cord, produces tachycardia, for example, as part of a fight or flight response to stress. We would argue that such a mode of action reflects much more the variety of responses that are observed on local application of GLP-1 or its analogues into specific brain areas, where effects on food intake, reward and addictive behavior, as well as on blood glucose, thermoregulation, and cardiovascular regulation, are reported dependent on the precise site of local application.

The obvious question that arises from these various quite disparate effects of GLP-1 within the brain is whether these effects represent simply different aspects of an integrated response to PPG neuron activation. Or do these diverse effects reflect the fact that several PPG neuron subpopulations exist with each tuned to a specific function? Randolph et al (24) demonstrated that PPG neurons innervate the PVT, PVN, and BNST collectively, and proposed similar groups of circuits could be possible for other part of the forebrain. At some overarching level GLP-1R activation within the CNS (and by extension PPG neuron activation) is linked to hypophagia, but when examining details, this hypophagia might occur in response to various reasons. It could be in response to restraint stress or to ingesting a very large meal or linked to a reduced reward assigned to the intake, or the hypophagia might occur for even different reasons. Given the extent of axonal projections from the PPG neurons in NTS and IRT toward the limbic system, combined with the abundancy of GLP-1R–expressing neurons in this area, one might expect a role of PPG neurons in reward-related behavior. Some insights have emerged through direct stimulation of PPG neurons. Activation of PPG neurons in NTS decreases nicotine intake, suggesting that PPG neurons have a direct effect on addiction/reward circuits (77). Applebey et al (78) in 2025 described the brainstem GLP-1 system as reducing motivation/reward-seeking behavior. These findings align with reduced reward- and food-seeking behavior in female, but not male, rats following both optogenetic and chemogenetic stimulation (79). Additionally, the hypoactive or reduced locomotor behavior observed in various studies of chemogenetic PPG activation (32, 38, 72) may be attributed to reduced food-seeking behavior or reduced motivation via the reward-based circuit. However, to confirm such hypotheses further investigation is necessary.

## Transcriptomic Profiling

A large dataset of transcriptomes of cells from the dorsal vagal nucleus might give a first insight into PPG neuron–expression profiles. Ludwig et al (80) in 2021 demonstrated that the *Gcg*-expressing neurons in the NTS represent a distinct population of glutamatergic neurons, characterized by high expression of *Lepr* (*30*) and lower expression of *Cck*. This dataset also confirmed that PPG neurons do not express, for example, *Glp1r*, *Gipr*, or *Mc4r* (28, 30, 41). The expression of *Lepr* on PPG neurons has also been demonstrated by us using single-nucleus RNA sequencing (60). However, the expression of *Lepr* was observed to be lower than the expression of *Htr2c.* In comparison to Ludwig et al (80), we also observed a slight, and possibly negligible, expression of *Mc4r* both with single-nucleus RNA sequencing and fluorescence in situ hybridization (60). In line with these findings, a 5-HT_2c_R agonist activated PPG neurons, but they were not required for MC4R agonist–induced hypophagia (60). This alignment with functional in vivo and ex vivo data supports the validity of transcriptomics data as a useful source for interrogating PPG neurons, with the potential to reveal other targetable pathways within these cells.

A comparison between transcriptomic data and histological analysis in the dorsal vagal complex described PPG neurons as glutamatergic NTS neurons that do not express *Phox2b* nor *Lmx1b*, an unusual feature of NTS-located neurons according to the authors, since most glutamatergic neurons in the NTS are positive for at least one of the two transcription factors (81). This finding validated the earlier observation that lentiviruses delivering constructs under the control of the artificial Phox2b promoter PRSx8 are not active in PPG neurons (10). Importantly, this finding affects the interpretation of results from a few publications that assumed Phox2b expression in PPG neurons (77, 82, 83). The finding that most, if not all, PPG neurons are glutamatergic also confirms immunocytochemical demonstration of *Slc17a6* (vGlut2) expression by PPG neurons both of the rat and mouse (9, 84). Additionally, GLP-1 and vGlut2 are located in the same varicosities within the brainstem (9). The functional importance of glutamate as cotransmitter was also tested by Liu et al (67). Knocking out vGlut2 abolished light-evoked glutamate release in the PVN but did not affect the concomitant PPG neuron–induced food-intake suppression, demonstrating that food-intake suppression by PPG neurons projecting to the PVN is independent of glutamate release.

Transcriptomic datasets of the brainstem and/or dorsal vagal complex helped to identify and characterize the PPG neurons as a distinct neuronal population, but not enough information is available from current datasets to resolve whether PPG neurons constitute a homogenous or heterogeneous population.

## Are Preproglucagon Neurons of Potential Translational Interest?

While most questions about the precise physiological role of PPG neurons remain unanswered, the aforementioned chemogenetic and optogenetic experiments have demonstrated the strong capacity of these neurons to reduce food intake when activated. Yet, as discussed earlier, removal of GLP-1 or GLP-1Rs does not elicit strong hyperphagia and excessive weight gain. This finding could be interpreted as a positive, because it implies that activation of these neurons leads to a reduction in food intake that is in addition to that achievable with targeting core appetite or satiation pathways. From this observation follows one pertinent question to be answered when considering the potential for obesity treatment: Is the significant reduction in food intake the only substantive consequence to be considered, or does activation of PPG neurons elicit adverse effects such as nausea, or even more severe effects such as anxiety and activation of the HPA axis? While these possibilities have not been conclusively excluded, studies from at least two laboratories found either no conditioned taste avoidance or no disrupted behavioral satiety sequence on chemogenetic activation of NTS PPG neurons in combination with the observed reduction in acute food intake, arguing against obligatory induction of nausea (32, 38). Similarly, one of these studies found no evidence for anxiety using elevated plus maze and open-field paradigms (32). On the other hand, a recent study activating NTS PPG neurons chemogenetically reported mild anxiety-like behavior, particularly in female mice (72).

Translational exploitation of GLP-1 release from PPG neurons could be achieved in two ways. The first would be to activate these neurons in vivo without the use of transgenic technologies and thereby elicit release of GLP-1 and cotransmitters from these neurons. A couple of recent studies serve as proofs of principle that this might be possible. Peripherally administered oxytocin acutely reduces food intake, and this effect is greatly diminished when PPG neurons are ablated (38). Similarly, the 5-HT_2C_ receptor agonist lorcaserin requires PPG neurons for its food intake–suppressive effect (60).

The second approach would be to selectively target brain GLP-1Rs, or specifically those that are reached by PPG neurons, but not those that are accessible to current peripherally administered GLP-1RAs. This approach would not use any cotransmitter released by the PPG neurons. At present it is not clear how relevant this difference is, and studies thus far have concluded that GLP-1 release is the crucial component of PPG neuron action for those instances investigated (67).

It is clear that a variety of pathways converge onto the reduction in food intake and ultimately body weight as the final desired outcome. All of these different pathways have components of CNS signaling. Peripherally administered GLP-1RAs reduce food intake by engaging central nervous pathways (85-87), though most likely not via GLP1-Rs located inside the BBB (88). In contrast, PPG neurons, which do not project to the periphery (14), act by engaging GLP-1Rs within the brain. Additionally, activation of other pathways, such as the central melanocortin system, reduces food intake. While the ultimate target of food-intake reduction is the same for all these circuits, there are differences in the overlap in the actual circuits. For example, food-intake suppression by the 5-HT_2C_ receptor agonist lorcaserin is prevented by the loss of either MC4 receptors or PPG neurons (60, 89, 90). In contrast, neither of these two manipulations affects the hypophagia elicited by GLP-1RAs (38, 60). Consequently, lorcaserin and liraglutide can be given as a combination treatment to achieve greater reduction in food intake than either drug alone (60).

## Conclusions

Recent years have seen a renewed interest in PPG neurons since their effect on eating is distinctly separate from that of clinical GLP-1 therapy, and the latest studies evaluating their activity in freely behaving mice finally enables detailed examination of their precise role under various physiological conditions. Such investigations will be crucial to determine whether PPG neurons and their GLP-1 release will become a valuable tool in clinical practice for obesity treatment or is linked to too many adverse effects such as stress or anxiety.

## Data Availability

No new data were generated or analysed in support of this article.

## Abbreviations

BBB: blood-brain barrier
BNST: bed nucleus of the stria terminalis
CCK-8: cholecystokinin
CNS: central nervous system
*Gcg*: glucagon
GLP-1: glucagon-like peptide-1
GLP-2: glucagon-like peptide-2
GLP-1R: glucagon-like peptide-1 receptor
GLP-1RA: glucagon-like peptide-1 receptor agonist
HPA: hypothalamic-pituitary-adrenal
IRES: internal ribosome entry site
IRT: intermediate reticular nucleus
mRNA: messenger RNA
NTS: nucleus tractus solitarii
PPG: preproglucagon
PVN: paraventricular nucleus of the hypothalamus
PVT: paraventricular nucleus of the thalamus
*Slc17a6*: gene that encodes vGlut2
vGlut2: vesicular glutamate transporter 2
YFP: yellow fluorescent protein
